# Early Antibiotic Use and Retinopathy of Prematurity: A Single-Center Retrospective Cohort Study

**DOI:** 10.1016/j.xops.2025.100919

**Published:** 2025-08-20

**Authors:** Jason Y. Zhang, Deborah S. Bondi, Max J. Hyman, Dimitra Skondra, Simmer Beniwal, John Moir, Sarah H. Rodriguez

**Affiliations:** 1 Pritzker School of Medicine, University of Chicago, Chicago, Illinois; 2 Department of Ophthalmology and Visual Science, University of Chicago, Chicago, Illinois; 3 Department of Pharmacy, University of Chicago Medicine Comer Children’s Hospital, Chicago, Illinois; 4 The Center for Health and the Social Sciences, University of Chicago, Chicago, Illinois; 5 Center for Research Informatics, University of Chicago, Chicago, Illinois; 6 Department of Pediatrics, University of Chicago, Chicago, Illinois

**Keywords:** Antibiotics, Gut dysbiosis, Gut–retina, Microbiome, Retinopathy of prematurity

## Abstract

**Objective:**

Retinopathy of prematurity (ROP) has been linked to neonatal sepsis, with antibiotic use suggested as a connection. Given the role of antibiotics in gut dysbiosis and the gut–retina axis, we assessed whether exposure to different antibiotic classes is associated with the incidence of treatment-necessary ROP.

**Design:**

Retrospective cohort study.

**Subjects:**

Preterm infants born at the University of Chicago Medicine and screened for ROP between January 2012 and December 2023.

**Methods:**

Retrospective analysis was performed to compare systemic antibiotic exposure within the first 2 months of life between infants with type 1 ROP (ie, required treatment) and those that did not require treatment. Multivariable adjustment included birth weight (BW), gestational age (GA), bronchopulmonary dysplasia (BPD), intraventricular hemorrhage, necrotizing enterocolitis (NEC), and neonatal sepsis. To reduce potential confounding by indication, propensity score matching was performed.

**Main Outcome Measures:**

Type 1 ROP by systemic exposure to antibiotic classes.

**Results:**

Seven hundred twenty infants were included, with 50 treated for ROP. The rate of systemic antibiotic use was 97.3% in the control group and 100.0% in infants with type 1 ROP. Infants with type 1 ROP showed higher rates of BPD (*P* < 0.001) and bacterial infection (*P* = 0.007). No association was noted with NEC. In multivariable regression, the odds of treatment were significantly greater for infants receiving cephalosporins, carbapenems, and monobactams (odds ratio [OR], 4.25; 95% confidence interval [CI], 1.74–10.4; *P* = 0.002), and an exploratory analysis suggested a dose-response per day prescribed (OR, 1.07; 95% CI, 1.03–1.11; *P* = 0.001). When restricted to BW <750 g or GA <27 weeks (N = 259, with 49 treated for ROP), these associations remained significant (OR, 3.87; 95% CI, 1.57–9.51; *P* = 0.003; per day prescribed: OR, 1.07; 95% CI, 1.03–1.11; *P* = 0.001). After propensity score matching for BW, GA, BPD, sepsis, and any bacterial infection, the average percent of infants who required treatment was 9.9 percentage points higher among those who received cephalosporins, carbapenems, and monobactams compared to those same infants if they had not received them (95% CI, 2.0–17.8 percentage points; *P* = 0.014).

**Conclusions:**

Early exposure to broad-spectrum antibiotic classes, particularly cephalosporins, carbapenems, and monobactams, may be associated with type 1 ROP.

**Financial Disclosure(s):**

The authors has no/the authors have no proprietary or commercial interest in any materials discussed in this article.

The introduction of antibiotics has played a significant role in reducing mortality in neonatal sepsis, with rates declining in high-income countries over the past few decades.^1^ However, simultaneously, the concern for a potential delayed diagnosis has led to frequent empirical use of antibiotics. This is evidenced by antibiotics being the most prescribed medication in the neonatal intensive care unit despite the incidence of culture-positive sepsis remaining low.^2^^,^^3^ Consequences of this trend have been well-documented, with prolonged antibiotic exposure associated with adverse outcomes in premature infants including bronchopulmonary dysplasia (BPD), necrotizing enterocolitis (NEC), fungemia, late-onset sepsis, antibiotic resistance, and overall morbidity.^4^, ^5^, ^6^, ^7^

These consequences potentially extend to retinopathy of prematurity (ROP) as well. Retinopathy of prematurity is a vasoproliferative disease of the retina, characterized by initial incomplete vascularization of the retina and followed by aberrant neovascularization. Increased incidence and severity of ROP has been linked to neonatal sepsis across several studies, which suggests prolonged antibiotic use as a potential mediator in this process.^8^, ^9^, ^10^, ^11^, ^12^ Furthermore, a Canadian neonatal network study revealed that in very-low-birth-weight infants without culture-proven sepsis or NEC, increased antibiotic use was associated with increased odds of stage 3 or higher ROP.^13^ Given this connection between antibiotics and ROP, independent of other neonatal pathology, we hypothesize that direct alterations in gut microbiome composition, or gut dysbiosis, may drive the association.

The purpose of this study is to compare exposure to different antibiotic classes among preterm infants with ROP requiring treatment and those who did not require treatment.

## Methods

### Study Design and Patient Selection

A single-center retrospective cohort study was performed at the University of Chicago Medicine. All preterm infants screened for ROP between January 2012 and December 2023 were included. The decision for ROP screening followed recommendations provided by the American Academy of Pediatrics (birth weight [BW] ≤1500 g, gestational age [GA] ≤30 weeks, or high-risk for ROP per neonatologist or pediatrician assessment).^14^ The requirement for obtaining informed consent from patients was waived given the retrospective design. The study adhered to the tenets of the Declaration of Helsinki and was approved by the Institutional Review Board of the University of Chicago (IRB 23-0739, approved 7/7/2023). The significance level was set at *P* < 0.05. All data on antibiotic exposures and comorbidities were obtained by the Center for Research and Informatics at the University of Chicago. Data analysis was conducted in Stata 18.0.

Infants with severe ROP requiring treatment were based on standard of care, following the Early Treatment for ROP guidelines for type 1 ROP (zone I with plus disease; zone I, stage 3; zone II, stage 2 or 3 with plus disease).^15^ These infants were identified by a list actively maintained by the senior author as standard of care to follow all treated infants. To examine exposure risk in the early microbiome, systemic exposure within the first 2 months of life was determined for the following medication classes: penicillins, “other beta lactam antibacterials” (ie, cephalosporins, carbapenems, and monobactams), aminoglycosides, “other antibacterial drugs” (ie, vancomycin, daptomycin, colistin, metronidazole, linezolid, nitrofurantoin, methenamine, and fosfomycin), and antifungals. As quinolone use was largely limited to topical application prior to ROP treatment (moxifloxacin eye drops), they were not included in the analysis. For infants who received treatment, charts were reviewed by a neonatal pharmacist to confirm that antibiotic exposure occurred prior to treatment. The full list of generic and brand name drugs included in each medication class is found in Supplemental Table 1 available at www.ophthalmologyscience.org.

The usual practice for empiric antimicrobial selection for management of possible sepsis in the neonatal intensive care unit at the University of Chicago Medicine is initiation of ampicillin and gentamicin. Ampicillin is substituted with vancomycin initially in infants with concern for late-onset sepsis who have a central venous or arterial catheter. Gentamicin is substituted with cefotaxime or ceftazidime in infants with a concern for meningitis or renal dysfunction. The typical empiric course, if infection is ruled out, is 36 to 48 h of antimicrobial treatment. Metronidazole is typically added for treatment of NEC. Most other antimicrobials, including fourth-generation cephalosporins, carbapenems, monobactams, and antifungals, are restricted and require approval for use after discussion with the Pediatric Infectious Diseases team. These restricted antimicrobials may be selected based on culture results, patient severity, or likely source of infection.

Data on demographics and comorbidities were also collected, including sex, GA, BW, BPD, intraventricular hemorrhage, NEC, neonatal sepsis, septic shock, bacteremia, pneumonia, urinary tract infection, meningitis, and candidiasis. Comorbidity data were determined by the diagnosis codes found in Supplemental Table 2 available at www.ophthalmologyscience.org.

### Statistical Analysis

Descriptive statistics were performed and compared cases requiring ROP treatment and controls not requiring treatment. Continuous variables, including GA and BW, were presented as medians (interquartile ranges) and compared by Wilcoxon rank sum tests. Categorical variables, including exposure to antibiotic classes, were presented as frequencies (percentages) and compared by Pearson chi-squared or Fisher exact tests.

Given the potential for confounding by indication (ie, infants who are sicker are more likely to receive antibiotics and treatment), univariate and multivariable logistic regression analyses were performed to assess whether exposure to different antibiotic classes was associated with odds of type 1 ROP. To avoid overfitting models, we created “any bacterial infection” as a combined variable including NEC, neonatal sepsis, septic shock, bacteremia, pneumonia, urinary tract infection, and meningitis. Based on significance in univariate models, multivariable models adjusted for BW, GA, BPD, and any bacterial infection for antibiotic exposures or candidiasis for the antifungal exposure.

To minimize the potential for selection bias by BW and GA, the analysis was repeated, including infants born at <27 weeks or with BW < 750 g. This subgroup definition was based on findings from the Early Treatment for ROP study in which 51.9% of infants with BW < 750 g and 45.5% of infants born at GA < 27 weeks required treatment.^16^ Analysis of the complementary subgroup (BW ≥ 750 g and GA ≥ 27 weeks) was not performed because only 1 treated infant met those criteria.

To further reduce potential confounding by indication, propensity score matching for BW, GA, BPD, sepsis, and any bacterial infection was performed in the full sample and subgroup. One-to-1 nearest-neighbor matching with replacement was applied, and we calculated the average treatment effect on the treated, which compared the risk of type 1 ROP between infants exposed to antibiotics and those same infants if they had not been exposed. Statistical balance of covariates was assessed with standardized differences and variance ratios (Supplemental Tables 3 and 4 available at www.ophthalmologyscience.org).

## Results

A total of 1508 infants were potentially eligible for the study, of whom 720 had complete antibiotic and comorbidity data (Table 1). Among these 720 infants, 50 (6.9%) received treatment for ROP. Compared to controls not requiring treatment, type 1 ROP cases showed younger GAs (*P* < 0.001), lower BWs (*P* < 0.001), and higher rates of BPD (*P* < 0.001) and any bacterial infection (*P* = 0.007). An association between ROP treatment and neonatal sepsis or candidiasis trended toward significance (neonatal sepsis: *P* = 0.06; candidiasis: *P* = 0.08). No association was noted with sex, intraventricular hemorrhage, or NEC.

**Table 1:** Demographics, Comorbidities, and Medication Exposures for Treated Retinopathy of Prematurity Cases and Untreated Controls

Characteristic	Untreated (N = 670)	Treated (N = 50)	*P* Value
Gestational age, weeks (median, IQR)	28.7 (26.9–30.3)	25.0 (24.3–25.4)	<0.001
Birth weight, grams (median, IQR)	1102.5 (840.0–1340.1)	627.5 (500.1–709.9)	<0.001
Sex (N, %)			0.88
Female	341 (50.9)	26 (52.0)	
Male	329 (49.1)	24 (48.0)	
Bronchopulmonary dysplasia (N, %)	72 (10.8)	16 (32.0)	<0.001
Intraventricular hemorrhage (N, %)	23 (3.4)	2 (4.0)	0.69
Necrotizing enterocolitis (N, %)	3 (0.5)	1 (2.0)	0.25
Neonatal sepsis (N, %)	18 (2.7)	4 (8.0)	0.06
Any bacterial infection (N, %)∗	41 (6.1)	8 (16.0)	0.007
Candidiasis (N, %)	30 (4.5)	5 (10.0)	0.08
Penicillins (N, %)	648 (96.7)	50 (100.0)	0.39
Other beta-lactam antibacterials (N, %)	209 (31.2)	43 (86.0)	<0.001
Days prescribed, median (IQR)†	1.0 (0.0–7.0)	8.0 (2.0–14.0)	<0.001
Aminoglycosides (N, %)	651 (97.2)	50 (100.0)	0.64
Other antibacterial drugs (N, %)	279 (41.6)	45 (90.0)	<0.001
Antifungals (N, %)	83 (12.4)	32 (64.0)	<0.001

IQR = interquartile range.

∗ Any bacterial infection includes necrotizing enterocolitis, neonatal sepsis, septic shock, bacteremia, pneumonia, urinary tract infection, and meningitis.

† The median (IQR) is among cases or controls who received other beta-lactam antibacterials.

On univariate analysis (Table 2), significant associations were noted with other beta-lactam antibacterials (odds ratio [OR], 13.55; 95% confidence interval [CI], 6.00–30.62; *P* < 0.001), other antibacterial drugs (OR, 12.61; 95% CI, 4.94–32.18; *P* < 0.001), and antifungals (OR, 12.57; 95% CI, 6.75–23.41; *P* < 0.001).

**Table 2:** Univariate and Multivariable Logistic Regression Analysis of Medication Classes for Treated Retinopathy of Prematurity Cases and Untreated Controls (N = 720)

Medication Class	Odds Ratio (95% Confidence Interval); *P* Value	
	Univariate Analyses	Multivariable Analysis
Other beta-lactam antibacterials∗	13.55 (6.00–30.62); *P* < 0.001	4.25 (1.74–10.41); *P* = 0.002
Per day prescribed∗	1.14 (1.09–1.18); *P* < 0.001	1.07 (1.03–1.11); *P* = 0.001
Other antibacterial drugs∗	12.61 (4.94–32.18); *P* < 0.001	2.47 (0.88–6.91); *P* = 0.09
Antifungals†	12.57 (6.75–23.41); *P* < 0.001	1.70 (0.78–3.73); *P* = 0.18

∗ Multivariable analysis adjusted for gestational age, birth weight, bronchopulmonary dysplasia, and any bacterial infection.

† Multivariable analysis adjusted for gestational age, birth weight, bronchopulmonary dysplasia, and candidemia.

Recognizing the role of supplemental oxygen in the development of ROP, we used BPD as a proxy for oxygen use and controlled for the variable on multivariable logistic regression. After adjusting for additional factors, including BW, GA, and any bacterial infection, other beta-lactam antibacterials remained significant (OR, 4.25; 95% CI, 1.74–10.41; *P* = 0.002), and an exploratory analysis suggested a dose-response per day prescribed (OR, 1.07; 95% CI, 1.03–1.11; *P* = 0.001). No association was found for other antibacterial drugs. These analyses were repeated including sepsis instead of any bacterial infection with no change in significance. No association was found for antifungals after adjusting for BW, GA, BPD, and candidemia.

Each infant exposed to other beta-lactam antibacterials (N = 252) was matched to a control after propensity score matching for BW, GA, BPD, sepsis, and any bacterial infection. The matched standardized differences of covariates in this model ranged from –0.066 to –0.015, and the matched variance ratios ranged from 0.88 to 1.30 (Supplemental Table 3). While the rate of treatment was the same before and after matching for infants exposed to other beta-lactam antibacterials as every exposed infant was matched (17.1%, 43/252), the rate of treatment increased from 1.5% (7/468) to 7.1% (18/252) for infants not exposed to other beta-lactam antibacterials. Accordingly, the average percent of infants who required treatment was 9.9 percentage points higher among those who received other beta-lactam antibacterials compared to those same infants if they had not received them (95% CI, 2.0–17.8 percentage points; *P* = 0.014).

To minimize the impact of BW and GA as potential confounders, we repeated the analysis after restricting infants to BW < 750 grams or GA < 27 weeks; Table 3). Birth weight and GA remained significantly lower in the type 1 ROP group. However, other comorbidities, including rates of sepsis and any type of infection were not different between cases and controls. On univariate analysis (Table 4), other beta-lactam antibacterials, other antibacterial drugs, and antifungals remained significantly associated with severe ROP. After adjusting for BW, GA, BPD, and any bacterial infection, the association between other beta-lactam antibacterials and type 1 ROP remained significant (OR, 3.87; 95% CI, 1.57–9.51; *P* = 0.003) and an exploratory analysis suggested a dose-response per day prescribed (OR, 1.07; 95% CI, 1.03–1.11; *P* = 0.001).

**Table 3:** Demographics, Comorbidities, and Medication Exposures for Treated Retinopathy of Prematurity Cases and Untreated Controls among Infants with Gestational Age < 27 Weeks or Birth Weight < 750 g

Characteristic	Untreated (N = 210)	Treated (N = 49)	*P* Value
Gestational age, weeks (median, IQR)	26.0 (24.9–26.7)	25.0 (24.3–25.4)	<0.001
Birth weight, grams (median, IQR)	732.6 (629.9–849.9)	625.1 (500.1–709.9)	<0.001
Sex (N, %)			0.65
Female	119 (56.7)	26 (53.1)	
Male	91 (43.3)	23 (46.9)	
Bronchopulmonary dysplasia (N, %)	51 (24.3)	16 (32.7)	0.23
Intraventricular hemorrhage (N, %)	8 (3.8)	2 (4.1)	1.00
Necrotizing enterocolitis (N, %)	2 (1.0)	1 (2.0)	0.47
Neonatal sepsis (N, %)	12 (5.7)	4 (8.2)	0.51
Any bacterial infection (N, %)∗	20 (9.5)	8 (16.3)	0.17
Candidiasis (N, %)	11 (5.2)	5 (10.2)	0.19
Penicillins (N, %)	209 (99.5)	49 (100.0)	1.00
Other beta-lactam antibacterials (N, %)	105 (50.0)	42 (85.7)	<0.001
Days prescribed, median (IQR)†	1.0 (0.0–7.0)	8.0 (3.0–14.0)	<0.001
Aminoglycosides (N, %)	210 (100.0)	49 (100.0)	NA
Other antibacterial drugs (N, %)	148 (70.5)	44 (89.8)	0.005
Antifungals (N, %)	64 (30.5)	32 (65.3)	<0.001

IQR = interquartile range; NA = not applicable.

∗ Any bacterial infection includes necrotizing enterocolitis, neonatal sepsis, septic shock, bacteremia, pneumonia, urinary tract infection, and meningitis.

† The median (IQR) is among cases or controls who received other beta-lactam antibacterials.

**Table 4:** Univariate and Multivariable Logistic Regression Analysis of Medication Classes for Treated Retinopathy of Prematurity Cases and Untreated Controls among Infants with Gestational Age < 27 Weeks or Birth Weight < 750 g (N = 259)

Medication Class	Odds Ratio (95% Confidence Interval); *P* Value	
	Univariate Analyses	Multivariable Analysis
Other beta-lactam antibacterials∗	6.00 (2.58–13.96); *P* < 0.001	3.87 (1.57–9.51); *P* = 0.003
Per day prescribed∗	1.09 (1.05–1.14); *P* < 0.001	1.07 (1.03–1.11); *P* = 0.001
Other antibacterial drugs∗	3.69 (1.40–9.74); *P* = 0.008	2.16 (0.77–6.07); *P* = 0.15
Antifungals†	4.29 (2.22–8.29); *P* < 0.001	1.84 (0.84–4.04); *P* = 0.13

∗ Multivariable analysis adjusted for gestational age, birth weight, bronchopulmonary dysplasia, and any bacterial infection.

† Multivariable analysis adjusted for gestational age, birth weight, bronchopulmonary dysplasia, and candidemia.

Each BW < 750 g or GA < 27 weeks infant exposed to other beta-lactam antibacterials (N = 147) was matched to a control after propensity score matching for BW, GA, BPD, sepsis, and any bacterial infection. The matched standardized differences of covariates in this model ranged from –0.087 to 0.137, and the matched variance ratios ranged from 0.92 to 1.59 (Supplemental Table 4). While the rate of treatment was the same before and after matching for infants exposed to other beta-lactam antibacterials as every exposed infant was matched (28.6%, 42/147), the rate of treatment increased from 6.3% (7/112) to 11.6% (17/147) for infants not exposed to other beta-lactam antibacterials. Accordingly, we found that the average percent of infants who required treatment was 23.8 percentage points higher among those who received other beta-lactam antibacterials compared to those same infants if they had not received them (95% CI, 6.5–41.2 percentage points; *P* = 0.007).

Finally, to capture as many exposed infants as possible, all analyses were repeated to include infants with exposure to antibiotics at up to 6 months of life. These results showed no difference in significance compared to the study population with antibiotic exposure at up to 2 months of life as presented above.

## Discussion

In this single-center retrospective cohort study, we demonstrate that early exposure to specific antibiotic classes, particularly cephalosporins, carbapenems, and monobactams, may be associated with treatment-necessary ROP (type 1 ROP). Many of the evaluated medication classes were associated with ROP on univariate analysis, though these associations are potentially complicated by the higher rate of infections among younger infants.^17^ Unique to cephalosporins, carbapenems, and monobactams, however, was the persistent significant association with type 1 ROP (1) in a subgroup of younger and smaller infants, (2) after multivariable adjustment including a covariate for any bacterial infection, and (3) in propensity score matching. Although undocumented infection with subsequent inflammation and poor growth may partially explain this relationship, a similar trend would have been expected across other antibiotic classes. The consistently significant relationship after adjustment and propensity matching, alongside the dose-response effect, suggest a notable association specific to this antibiotic class, which may be due to its broad-spectrum coverage.

Multiple antibiotic classes, including cephalosporins, have been shown to persistently reduce microbial diversity and increase antibiotic resistance burden in healthy adults.^18^^,^^19^ The particularly broad-spectrum coverage of cephalosporins, carbapenems, and monobactams may lead to significant alterations in the gut microbiome, impacting overall health. Gut dysbiosis secondary to cephalosporin administration has been shown to accelerate pulmonary endothelial barrier dysfunction in mice with *Streptococcus pneumoniae*.^20^ Meanwhile, exposure to broad-spectrum antibiotics has also been shown to be associated with greater odds of new-onset age-related macular degeneration compared to narrow-spectrum antibiotics.^21^ Similarly, while we cannot rule out that sicker infants may be more likely to receive this class of antibiotics in our study population, there is evidence supporting an association between ROP and broad-spectrum antibiotics.^13^

The impact of antibiotics on the neonatal gut microbiota has been well-documented in neonatology. A recent prospective study revealed that exposure to perinatal antibiotics altered gut microbiome composition at the phylum level at 12 months of age.^22^ Notably, this effect was dose-dependent and strongest when exposure occurred during the intrapartum or immediate postpartum phases. Such impacts on the early microbial environment may significantly affect the risk for various neonatal pathologies, including BPD and NEC.^23^^,^^24^ While the precise mechanisms behind these associations remain unclear, it is hypothesized that inflammation through toll-like receptor 4, tumor necrosis factor alpha, and interleukin 6 may contribute to the pathologies’ clinical progressions.^25^^,^^26^

Similar connections between early gut dysbiosis and ROP have been proposed as well. Our group previously identified significant perturbations in gut microbiome composition among preterm infants at 28 weeks’ postmenstrual age who later developed type 1 ROP. Infants with ROP displayed enrichment in Enterobacteriaceae populations alongside decreases in several metabolic pathways, including oxidative phosphorylation and amino acid metabolism.^27^ Other studies have revealed similar changes among preterm infants with ROP, including reductions in alpha diversity and significant enrichments in *Staphylococcus*—a species that has been suggested to disrupt tissue revascularization.^28^^,^^29^ Given these findings, probiotic supplementations have been proposed and evaluated as a potential option to attenuate the risk of ROP, with varying degrees of success. While a prior meta-analysis found no significant effect,^30^ a more recent study revealed a protective role for probiotics against ROP.^31^

There are several limitations in our study. While exposure to different antibiotic classes was identified during data collection, the degree of exposure was determined only for other beta-lactam antibacterials as this antibiotic class remained significant on multivariable logistic regression. In addition, the time frame for antibiotic exposure may have included exposure to antibiotics at different postmenstrual ages when comparing treated to untreated infants. However, all charts were reviewed for treated infants to confirm that exposure to systemic antibiotics occurred prior to treatment. As a result, this study design may dilute the significance of an association by capturing more antibiotic exposure among untreated infants. Similarly, as data were collected in part by diagnosis codes, our analysis may be affected by undercoding of potential confounding variables. However, this would be expected to uniformly impact the analysis for every antibiotic class.

In addition, given the single-center design, the external validity of our observations may be limited due to incomplete records, regional patient demographics, and distinct clinical practices. However, our results may help narrow down antibiotic classes that warrant further investigation at other institutions. Furthermore, although multivariable regression was performed, there may be additional confounding factors that remained unaccounted for in the analysis, such as poor postnatal weight gain and serum insulin-like growth factor 1 levels.^32^^,^^33^ Nevertheless, given our adjustments for significant confounders including neonatal sepsis and NEC, we believe that these findings warrant further investigation. Finally, this study cannot prove or establish causality as the effects of antibiotics may not be mediated by gut dysbiosis but other confounding variables. For instance, after restricting analysis to BW < 750 g or GA < 27 weeks, a significant difference in BW and GA remained between the untreated and treated groups (Table 3), suggesting that the treated group may have been more vulnerable and selectively received antibiotics.

Antimicrobial stewardship interventions have been developed to reduce antibiotic use, resulting in substantial reductions in treatment duration among newborns without culture-positive sepsis.^34^^,^^35^ Together, our study further supports these interventions in the context of mitigating ROP risk and development. Although it remains unclear how broad-spectrum beta-lactams specifically contribute to the development of treatment-necessary ROP, its significant relationship after controlling for neonatal sepsis and NEC suggests that gut dysbiosis may play a role in this process. Further studies are needed to confirm and elucidate the underlying mechanisms of this newly described association.
