# Supplementary material for: Early Antibiotic Use and Retinopathy of Prematurity: A Single-Center Retrospective Cohort Study
Source: Ophthalmol Sci. 2025 Aug 20;6(1):100919. doi: 10.1016/j.xops.2025.100919 (PMC12548081; doi:10.1016/j.xops.2025.100919)
Supplement: Supplemental Table 1 [file mmc1.docx]

**Supplemental Table 1: Generic and Brand Drug Names for Medication Classes**

| **Medication Class** | **Generic Names (contains)** | **Brand Names (contains)** |
| --- | --- | --- |
| Penicillins, betalactamase inhibitors, and combinations | amoxicillin, ampicillin, bacampicillin, piperacillin, ticarcillin, carbenicillin, dicloxacillin, nafcillin, oxacillin, cloxacillin, penicillin | Amoxicot, Amoxil, DisperMox, Moxatag, Moxilin, Trimox, Unasyn, Ampi, Omnipen, Penglobe, Principen, Spectrobid, Zosyn, Tazocin, Pipracil, Timentin, Geocillin, Dycill, Dynapen, Nallpen, Unipen, Bactocil, Cloxapen, Tegopen, Amoxil, Bactocill, Bicillin, Cloxapen, Crysticilli, Dynapen, Geocillin, Nafcil, Pfizerpen, Pipracil, Principen, Staphcillin, Ticar, Veetids |
| Other beta lactam antibacterials | cefadroxil, cefazolin, cephalexin, cefotetan, cefoxitin, cefaclor, cefprozil, cefuroxime, cefdinir, cefditoren, cefixime, cefotaxime, cefpodoxime, ceftazidime, ceftibuten, ceftriaxone, cefepime, ceftaroline, aztreonam, imipenem, meropenem, ertapenem, doripenem | Duricef, Ancef, Kefzol, Zolicef, Bio-Cef, Keflex, Panixine, Cefotan, Mefoxin, Ceclor, Raniclor, Cefzil, Ceftim, Omnicef, Spectracef, Suprax, Claforan, Vantin, Fortaz, Tazicef, Fortum, Cedax, Rocephin, Maxipime, Teflaro, Azactam, Primaxin, Merrem, INVanz, Doribax |
| Aminoglycosides | paromomycin, amikacin, plazomicin, tobramycin, neomycin, kanamycin, gentamicin | Humatin, Amikin, Zemdri, Nebcin, Neo-Fradin, Kantrex, Garamycin |
| Other antibacterial drugs | vancomycin, daptomycin, colistin, metronidazole, linezolid, nitrofurantoin, methenamine, fosfomycin | Firvanq, Vancocin, Vancoled, Cubicin, Dapzura, Coly-Mycin, Flagyl, Zyvox, Furadantin, Macrodantin, Hiprex, Mandelamine, Urex, Monurol |
| Antifungals | amphotericin, fluconazole | Fungilin, Fungizone, Abelcet, AmBisome, Fungisome, Amphocil, Amphotec, Halizon, Azocan, Diflucan, Canesten |
