# Supplementary material for: Early Antibiotic Use and Retinopathy of Prematurity: A Single-Center Retrospective Cohort Study
Source: Ophthalmol Sci. 2025 Aug 20;6(1):100919. doi: 10.1016/j.xops.2025.100919 (PMC12548081; doi:10.1016/j.xops.2025.100919)
Supplement: Supplemental Table 2 [file mmc2.docx]

**Supplemental Table 2: Diagnosis Codes for Comorbidities**

| **Comorbidity** | ***ICD-9*** | ***ICD-10*** |
| --- | --- | --- |
| Bronchopulmonary dysplasia | 770.7 | P27.1 |
| Intraventricular hemorrhage | 772.1x | P52.0, P52.1, P52.21, P52.22, P52.3 |
| Necrotizing enterocolitis | 777.5x | K55.3x |
| Neonatal sepsis | 771.81, 995.91, 995.92 | P36.x, R65.20 |
| Septic shock | 785.52 | R65.21 |
| Bacteremia | 771.83 | R78.81 |
| Pneumonia | 003.22, 011.6x, 055.1, 073.0, 112.4, 115.05, 136.3, 480.x, 481.x, 482.x, 483.x, 484.x, 485.x, 486.x, 487.0, 488.11, 770.0 | A01.03, A02.22, A37.01, A37.11, A37.81, A37.91, A50.04, A54.84, B01.2, B05.2, B06.81, B25.0, B37.1, B39.2, B44.0, B59, B77.81, J09.x1, J10.00, J10.01, J10.08, J11.08, J12.x, J13, J14, J15.x, J16.x, J17, J18.x, P23.x |
| Urinary tract infection | 771.82 | P39.3 |
| Meningitis | 003.21, 013.0x, 036.0, 047.x, 049.0, 049.1, 053.0, 054.72, 072.1, 090.42, 091.81, 094.2, 098.82, 100.81, 112.83, 114.2, 115.01, 155.11, 115.91, 320.x, 321.x, 322.x | A01.01, A02.21, A17.0, A20.3, A27.81, A32.11, A39.0, A42.81, A50.41, A51.41, A52.13, A54.81, A69.21, A87.x, B00.3, B01.0, B02.1, B05.1, B06.02, B26.1, B27.02, B27.12, B27.82, B27.92, B37.5, B38.4, B57.41, G00.x, G01, G02, G03.x |
| Candidiasis | 112.x, 771.7 | B37.x, P37.5 |
