# Supplementary material for: Early Antibiotic Use and Retinopathy of Prematurity: A Single-Center Retrospective Cohort Study
Source: Ophthalmol Sci. 2025 Aug 20;6(1):100919. doi: 10.1016/j.xops.2025.100919 (PMC12548081; doi:10.1016/j.xops.2025.100919)
Supplement: Supplemental Table 3 [file mmc3.docx]

**Supplemental Table 3: Statistical Balance Before and After Propensity Score Matching for Multivariable Logistic Regression Analysis of Other Beta Lactam Antibacterials**

| **Covariate** | **Before Matching (N=720)** | | | | | | |
| --- | --- | --- | --- | --- | --- | --- | --- |
|  | **Mean** | | **Standardized**  **Difference** | **Variance** | | **Variance**  **Ratio** |  |
|  | **Exposed to Other Beta**  **Lactam Antibacterials**  **(N=252)** | **Not Exposed to Other Beta**  **Lactam Antibacterials**  **(N=468)** |  | **Exposed to Other Beta**  **Lactam Antibacterials**  **(N=252)** | **Not Exposed to Other Beta Lactam Antibacterials**  **(N=468)** |  |  |
| Gestational Age, Weeks | 27.04592 | 29.04365 | -0.8116254 | 6.339943 | 5.777009 | 1.097444 |  |
| Birth Weight, Grams | 894.3975 | 1142.159 | -0.8074299 | 112328.7 | 75987.63 | 1.478249 |  |
| Bronchopulmonary Dysplasia | 0.2103175 | 0.0747863 | 0.3944735 | 0.1667457 | 0.0693415 | 2.404703 |  |
| Neonatal Sepsis | 0.0714286 | 0.008547 | 0.3245397 | 0.0665908 | 0.0084921 | 7.841495 |  |
| Any Bacterial Infection | 0.1388889 | 0.0299145 | 0.3990409 | 0.1200753 | 0.0290818 | 4.128881 |  |
|  |  |  |  |  |  |  |  |
|  | **After Matching (N=504)** | | | | | | |
|  | **Mean** | | **Standardized**  **Difference** | **Variance** | | **Variance**  **Ratio** |  |
| **Covariate** | **Exposed to Other Beta**  **Lactam Antibacterials**  **(N=252)** | **Not Exposed to Other Beta**  **Lactam Antibacterials**  **(N=252)** |  | **Exposed to Other Beta**  **Lactam Antibacterials**  **(N=252)** | **Not Exposed to Other Beta Lactam Antibacterials**  **(N=252)** |  |  |
| Gestational Age, Weeks | 27.04592 | 27.18934 | -0.0580362 | 6.339943 | 5.874568 | 1.079219 |  |
| Birth Weight, Grams | 894.3975 | 908.3135 | -0.0441551 | 112328.7 | 86325.15 | 1.301228 |  |
| Bronchopulmonary Dysplasia | 0.2103175 | 0.2261905 | -0.0383586 | 0.1667457 | 0.1757257 | 0.9488979 |  |
| Neonatal Sepsis | 0.0714286 | 0.0753968 | -0.0151852 | 0.0665908 | 0.0699899 | 0.9514344 |  |
| Any Bacterial Infection | 0.1388889 | 0.1626984 | -0.06644 | 0.1200753 | 0.1367704 | 0.8779332 |  |
