# Supplementary material for: Early Antibiotic Use and Retinopathy of Prematurity: A Single-Center Retrospective Cohort Study
Source: Ophthalmol Sci. 2025 Aug 20;6(1):100919. doi: 10.1016/j.xops.2025.100919 (PMC12548081; doi:10.1016/j.xops.2025.100919)
Supplement: Supplemental Table 4 [file mmc4.docx]

**Supplemental Table 4: Statistical Balance Before and After Propensity Score Matching for Multivariable Logistic Regression Analysis of Other Beta Lactam Antibacterials among Infants with Gestational Age <27 Weeks or Birth Weight <750 Grams**

| **Covariate** | **Before Matching (N=259)** | | | | | |
| --- | --- | --- | --- | --- | --- | --- |
|  | **Mean** | | **Standardized**  **Difference** | **Variance** | | **Variance**  **Ratio** |
|  | **Exposed to Other Beta**  **Lactam Antibacterials**  **(N=147)** | **Not Exposed to Other Beta**  **Lactam Antibacterials**  **(N=112)** |  | **Exposed to Other Beta**  **Lactam Antibacterials**  **(N=147)** | **Not Exposed to Other Beta**  **Lactam Antibacterials**  **(N=112)** |  |
| Gestational Age, Weeks | 25.41691 | 25.95663 | -0.3740048 | 2.647892 | 1.517132 | 1.745328 |
| Birth Weight, Grams | 669.6403 | 818.002 | -0.8105983 | 24921.45 | 42076.68 | 0.5922866 |
| Bronchopulmonary Dysplasia | 0.3061224 | 0.1964286 | 0.2539602 | 0.2138664 | 0.1592664 | 1.342822 |
| Neonatal Sepsis | 0.1020408 | 0.0089286 | 0.4139658 | 0.0922561 | 0.0089286 | 10.33268 |
| Any Bacterial Infection | 0.1632653 | 0.0357143 | 0.4345735 | 0.1375454 | 0.034749 | 3.958252 |
|  |  |  |  |  |  |  |
|  | **After Matching (N=294)** | | | | | |
|  | **Mean** | | **Standardized**  **Difference** | **Variance** | | **Variance**  **Ratio** |
| **Covariate** | **Exposed to Other Beta**  **Lactam Antibacterials**  **(N=147)** | **Not Exposed to Other Beta**  **Lactam Antibacterials**  **(N=147)** |  | **Exposed to Other Beta**  **Lactam Antibacterials**  **(N=147)** | **Not Exposed to Other Beta**  **Lactam Antibacterials**  **(N=147)** |  |
| Gestational Age, Weeks | 25.41691 | 25.43635 | -0.013232 | 2.647892 | 1.667259 | 1.588171 |
| Birth Weight, Grams | 669.6403 | 664.2404 | 0.0335101 | 24921.45 | 27012.29 | 0.922597 |
| Bronchopulmonary Dysplasia | 0.3061224 | 0.3469388 | -0.0868245 | 0.2138664 | 0.2281241 | 0.9375 |
| Neonatal Sepsis | 0.1020408 | 0.0884354 | 0.0462035 | 0.0922561 | 0.0811667 | 1.136625 |
| Any Bacterial Infection | 0.1632653 | 0.1156463 | 0.1373162 | 0.1375454 | 0.1029727 | 1.335747 |

Note: The ‘N’ of those not exposed to other beta lactam antibacterials increased after matching because matching with replacement was applied.
